# ESCAP Expert Paper: New developments in the diagnosis and treatment of adolescent anorexia nervosa—a European perspective

**DOI:** 10.1007/s00787-015-0748-7

**Published:** 2015-07-31

**Authors:** Beate Herpertz-Dahlmann, Annemarie van Elburg, Josefina Castro-Fornieles, Ulrike Schmidt

**Affiliations:** Department of Child & Adolescent Psychiatry, Psychosomatics and Psychotherapy, University Clinics, RWTH Aachen, Neuenhofer Weg 21, 52074 Aachen, Germany; Department of Social Sciences, Rintveld, Center for Eating Disorders, Altrecht Mental Health Institute, Utrecht University, Utrecht, The Netherlands; Department of Child & Adolescent Psychiatry, Neurosciences Institute, Hospital Clinic of Barcelona, CIBERSAM, IDIBAPS, University of Barcelona, Barcelona, Spain; Institute of Psychiatry, Psychology and Neuroscience, King’s College London, London, UK

**Keywords:** Anorexia nervosa, Adolescence, Treatment, European guidelines, Review

## Abstract

Anorexia nervosa is a potentially life-threatening disorder with a typical onset in adolescence and high rates of medical complications and psychiatric comorbidity. This article summarizes issues relating to classification in DSM-5 and presents a narrative review of key evidence-based medical and behavioral interventions for adolescent AN and subthreshold restricting eating disorders, mainly, but not exclusively published between 2012 and 2014. In addition, it systematically compares the clinical guidelines of four European countries (Germany, Spain, The Netherlands, and United Kingdom) and outlines common clinical practice, in relation to treatment settings, nutritional rehabilitation, family-oriented and individual psychotherapy, and psychopharmacological treatment. With the exception of family-based treatment, which is mainly evaluated and practiced in Anglo-American countries, the evidence base is weak, especially for medical interventions such as refeeding and pharmacological intervention. There is a need for common European research efforts, to improve the available evidence base and resulting clinical guidance.

## Introduction

Eating is a basic need that is taken for granted by most human beings. However, disorders of eating are severe mental disorders that are listed among the most common chronic illnesses among youth [1] and are often associated with high personal, familial, and societal costs. In anorexia nervosa (AN), the mortality risk is higher than for other serious diseases of adolescence, such as asthma, type 1 diabetes, or any other psychiatric disorder [2].

There is some evidence that the age of onset of AN has been decreasing in recent decades [3, 4] and that childhood and adolescent AN are on the rise [5]. However, despite the seriousness and high chronicity of the illness, it remains a relatively small research area with significantly fewer publications than in other fields of mental disorders. Research on AN treatment has been particularly neglected. Only 12 papers among the top 100 cited papers published in the AN field address therapeutic strategies [6], although there have been some scientific advances that may be translated into new treatment methods. It is the aim of this article to present a clinically relevant overview of recent progress in the treatment of childhood and adolescent AN to support early intervention and effective treatment strategies in this serious disorder of youth. New developments in the concept and definition of eating disorders, especially of AN, as effectuated in DSM-5 [7] and the beta draft of ICD-11 (http://apps.who.int/classifications/icd11/browse/f/en), are presented.

## Method

In January 2015, we conducted a narrative review of evidence-based medical and psychological treatments for AN and subthreshold restricting eating disorders in youth with an emphasis on European clinical research, but also including key international contributions. We especially, but not exclusively searched for clinical research updates from the last 2 years. In addition, we systematically compared the clinical guidelines of four European countries: Germany (German S3-Guidelines, [8]), the Netherlands [9], Spain (Clinical Practice Guideline for Eating Disorders, [10] and United Kingdom (NICE [11]). In addition, two recent reviews of treatment in AN were consulted [12, 13].

## Classification and transition from DSM-IV to DSM-5

An important goal of DSM-5 [7] was the reduction of the residual diagnosis “Eating disorder not otherwise specified” (EDNOS) by, i.a., lowering the threshold for the diagnosis of the “classic” eating disorders AN and bulimia nervosa (BN) and by introducing binge eating disorder (BED) as a distinct diagnosis. A major change in diagnostic criteria of AN from DSM-IV to DSM-5 was the omission of the amenorrhea criterion. This issue is of some importance for our patients because the amenorrhea item is not appropriate for premenarchal girls, adolescents on hormonal contraceptives, and males. Several studies have demonstrated that females who were still menstruating but fulfilled all other criteria for AN closely resemble those who also have amenorrhea [14, 15].

For child and adolescent psychiatrists, the weight item of the diagnostic criteria (criterion A, [7]) is more distinct in referring to pathological vs. “significantly low weight in context of […] physical health”, e.g., in referring to the effects of starvation. Underweight must be judged in the “context of age, sex, developmental trajectory and physical health” [7], which is, however, still unspecific for the somatic changes typical of AN-associated starvation. In addition to the rate of weight loss, the NICE and Spanish guidelines point to “objective physical signs” and “additional appropriate laboratory tests” for diagnosing AN, especially in children and adolescents. The German and Dutch guidelines even recommend specific physical and laboratory examinations. For children and adolescents, DSM-5 gives no standard measure for the weight criterion. “Guidance regarding how to judge whether a weight is significantly low” [16] is provided only for adults in DSM-5 [“a body mass index (BMI), calculated as weight in kilograms divided by height in m^2^, lower than 18.5, which corresponds to the 10^th^ BMI percentile in US and European adult populations”]. To standardize the weight criterion for youth in Europe, we would propose the 10th BMI age percentile as a sensible weight threshold in minors, which is already used in Germany (German guidelines, [8]) and by international clinicians [17] (for a more detailed discussion and review see [18]). In addition, it would be reasonable to establish severity criteria according to BMI percentiles (mild, moderate, severe, and extreme, probably corresponding to the 10th, 3rd, 1st, and less than 1st percentile, respectively) similar to those formulated in adult AN [14].

Recent research has reported objective and subjective stigmatization of individuals with eating disorders [19, 20], which is consistently associated with more damage to well-being and delayed enrolment in treatment. DSM-IV items that implied a deliberate attitude of the patient and willful actions (such as “refusal to maintain body weight at or above a minimally normal weight for age and height” or “denial of the seriousness of low body weight”) were replaced by more non-judgmental formulations such as “restriction of energy intake relative to requirements” or “persistent lack of recognition of the seriousness of the current low body weight”.

Moreover, the DSM-IV criteria “intense fear of gaining weight” as well as “a distortion of body image [21] are rarely reported by children and young adolescents and likely depend on the development of abstract reasoning, culture, and the stage of the disease. Thus, clinicians treating younger patients may rely on the clinical symptom “persistent behavior that interferes with weight gain” (DSM-5 criterion B), which is much more easily detectable by caregivers and professionals. The influence of cultural attitudes on body dissatisfaction, especially in Asian countries, was emphasized in several recent studies (for a review, see [22]).

DSM-5—similar to DSM-IV—distinguishes between the restricting and binge eating/purging subtypes of AN. In the restricting type, weight loss is primarily accomplished by fasting and/or excessive exercising; in the binge eating/purging type, restrictive eating is interrupted by binging and purging, only binging or only purging (such as self-induced vomiting, laxative abuse, or the use of other weight loss-inducing medications).

In DSM-5, atypical AN is subsumed under “Other Specified Feeding or Eating Disorders”. An eating disorder is classified under this category if it does not fulfill all diagnostic criteria for AN but still causes “clinically significant distress or impairment” in various types of functioning. In most cases, a diagnosis of atypical AN is used if the weight criterion is not fulfilled.

In a recent study of adults from the US, no significant differences between AN and subthreshold AN were found based on self-report or interview measures; the only difference was that participants with AN reported higher rates of binge eating and purging compared with those with subthreshold AN and more somatic sequelae, such as lower white blood cell counts [23].

The preliminary criteria for AN published in the beta draft version of ICD-11 are similar to those in DSM-5 (http://apps.who.int/classifications/icd11/browse/f/en). Analogous to DSM-5, the amenorrhea criterion was omitted. However, the weight criterion for children and adolescents is more specific in ICD-11 than in DSM-5. In contrast to a BMI threshold of 18.5 kg/m^2^ in adults, which corresponds to the 10th BMI percentile of the general population, an age-based BMI *under the 5th percentile* is defined as the threshold weight for children and adolescents. In the international survey by Cole et al. [24] three cut-offs at age 18 to define underweight are recommended: a BMI of 18.5 for grade 1 thinness, a BMI of 17 for grade 2 and a BMI of 16 for grade 3 thinness. Cole et al. propose a BMI of 17 at age 18 (which roughly corresponds to the 5th percentile) as a suitable threshold to use as the basis for providing age and sex-specific cut-off points for a definition of thinness in children and adolescents. However, as grade 1 thinness is used for defining the weight threshold for AN in the new classification system of DSM-5, it is difficult to understand why one would want to choose a lower BMI threshold for minors in ICD-11. AN-associated underweight in childhood and adolescence may have even more severe health effects than in adults, especially on growth and development. ICD-11 differentiates between AN with dangerously low body weight (<0.1 BMI percentile) and significantly low body weight (<5th percentile) in this younger age group.

## Epidemiology and changes in prevalence from DSM-IV to DSM-5

The majority of epidemiological surveys report that the highest incidence of AN is found in 15–19-year-old females, with approximately 40 % of all new cases appearing during this stage of life [5, 25]; for more details, see [26]. In a recent UK study based on a primary care register [25], an AN incidence rate of 47.5/100,000 15–19-year-old females/year (according to DSM-IV) was found for the year 2009. Incidence rates for AN in this age group remained stable between 2000 and 2009; however, there was a steady increase in the incidence of EDNOS during that time period (note, however, that the diagnosis of EDNOS includes atypical AN and other restrictive eating disorders). This incidence rate for AN was lower than the 109.2/100,000 population/year in a Dutch study using a primary care sample by the Hoek group [27]. Furthermore, in the UK study, 24/100,000 girls had an onset of AN during the age range of 10–14 years. In children between 5 and 12 years of age, the incidence of AN in the UK was estimated at 1.09, with a clear relationship between prevalence and increasing age [4].

Recent studies have stated that prevalence rates for eating disorders differ when using the revised version of DSM-5 criteria. When a Portuguese sample of high school and university female students (*n* = 3048) was reclassified according to DSM-5 criteria, the point prevalence rates were as follows: DSM-IV: 0.59; DSM-5 (with a weight threshold of <17.5): 0.69; and DSM-5 (with a weight threshold of <18.5): 0.95 [28]. In a very recent Dutch community study, the point prevalence of AN was 1.2 in females (and thus very similar to the UK rates), whereas the lifetime prevalence rate for 19-year-old females was 1.7 according to the DSM-5 [29].

### Incidence and prevalence rates in males

In the UK study based on primary care data, the incidence for 15–19-year-old males was 3.8/100,000 person/years, which was—similar to girls—also the peak age of incidence for AN. In contrast to females, the peak age of onset of EDNOS for males was 10–14 years [25].

The point and lifetime prevalence rates for 19-year-old males were 0.1 in the Dutch community study delineated above [29] and were thus very similar to those of a former investigation described by van Son et al. [27].

## Treatment

Most European and international guidelines have emphasized a multidisciplinary and multimodal approach addressing the medical, nutritional, and psychological needs of the affected individual (NICE guidelines, Spanish, Dutch and German guidelines; APA [30]). However, with the exception of outpatient family-based therapy (FBT), there is a dearth of controlled studies on the treatment of adolescent and childhood AN; hence, most recommendations are still based on mainstream clinical opinion with little empirical standing. This observation holds true for the choice of treatment setting and the treatment modality.

### Treatment settings

Adolescents with AN are treated in a variety of settings, the most important being outpatient treatment, day patient/partial hospital treatment, inpatient child and adolescent psychiatric treatment, and inpatient pediatric treatment. The use of various settings often depends on the health care system of the individual country rather than on scientific evidence.

### Inpatient treatment (IP)

While NICE guidelines recommend outpatient treatment for most patients with AN, the authors make a special modification for young patients (“the need for IP and the need for urgent weight restoration should be balanced alongside the educational and social needs of the young person”). Nevertheless, in the UK, admission rates for AN have increased substantially in recent years. From 2012 to 2013, there was a national rise of 8 % in the number of admissions to hospital for an eating disorder in comparison to the year before, which had already seen an increase. Adolescent girls with AN accounted for the vast majority of these admissions. In addition, the length of hospital stays increased substantially during the last decade (http://www.hscic.gov.uk/article/3880/Eating-disorders-Hospital-admissions-up-by-8-per-cent-in-a-year).

There are also increasing admission rates for young people with AN, especially children, in Germany [31]. In Germany, IP remains the treatment of choice for most children and adolescents with AN, because, among other reasons, many outpatient therapists do not dare to manage a young starved patient. Dutch guidelines propose a “stepped care model” offering the “least burdensome, cheapest and shortest treatment possible to the patient”, but for AN, “the lower levels of stepped care should not be applied”. In the US, eating disorders (without differentiating between subtypes) constitute the ninth most common mental disorder in inpatient national hospitals [32].

Different health care politics lead to different clinical populations being admitted to hospital. In a study from the UK, a continuous drop from a yearly average admission BMI of 14.1 kg/m^2^ in 2007 to 13.6 kg/m^2^ in 2009 was observed in adults with AN, which was related to a significantly higher likelihood of readmission within 1 year [33]. As far as we know, there are no comparable data for adolescents. However, in a recent study including two inpatient eating disorder units for adolescents in the UK, BMI in 13–17-year-olds was only slightly lower than that of a large German trial [34, 35] (see below). In Germany, a small but significant increase between 2000 and 2009 in the age-adjusted BMI percentile and absolute BMI in a childhood and adolescent sample was found [36, 35].

Only one randomized controlled trial (RCT) from the UK (*n* = 170 adolescents with AN) compared IP with two different forms of outpatient care (a specialized eating disorder outpatient treatment with emphasis on CBT and treatment as usual in child and adolescent mental health services) [37]. Although participants improved over the two-year follow-up period, there were no differences in clinical outcomes between the three groups. The findings of this study are difficult to interpret because treatment adherence differed substantially between groups, with poor treatment uptake in the IP treatment arm. In another recent RCT (*n* = 82) from Australia, adolescent patients were randomized either to hospitalization for medical stabilization (mean duration: 3 weeks, mean  %EBW at discharge: 84) or for weight restoration (mean duration: 5 weeks, mean %EBW at discharge: 92), thus comparing short and slightly longer inpatient treatment. Both treatment options were followed by outpatient manualized family-based treatment. Primary outcome was the number of days spent in hospital following the first admission. At the 12-month follow-up, there were no significant differences between groups. However, the analysis did not take into account the cluster randomization of the trial nor the distribution of the primary outcome variable (the majority of patients had no readmissions), thus no strong conclusions can be drawn on the comparative merits of these treatment approaches [38].

In the study mentioned above [34], data from patients admitted to 14 UK inpatient treatment units (*n* = 12 for adults, *n* = 2 for adolescents) were collected. In the adolescent subgroup, the change in BMI was twice as large as that in the adult sample; there was also a more pronounced amelioration in mood and quality of life. In contrast to the adults, adolescents’ “confidence to change” as assessed by a general scale of motivation to change also increased during treatment [34]. This is in line with the results of a study carried out in Spain, in which adolescent patients significantly improved in BMI, eating attitudes, depressive symptomatology, and motivation to change during inpatient treatment [39].

In the US, data collected by the National Eating Disorders Quality Improvement Collaborative were used to evaluate adolescent medicine-based eating disorder programs. Program outcomes did not differ significantly, although the proportion of inpatient treatment used in these programs varied substantially [40].

The European guidelines studied here agree on several criteria for inpatient care in adolescent AN (Table 1). However, some of the recommendations might be judged controversial. While we deem failure to improve in outpatient treatment as a reason for considering admission to hospital, Gowers et al. (2007) argued that his studies, comparing inpatient and outpatient treatment lend little support to a transfer from outpatient to inpatient care in the sense of a stepped care approach. However, in his study on clinical effectiveness of different treatment settings (2007) less than 50 % of the patients randomized to inpatient care actually received this treatment for at least 4 weeks, so that the results of this study might be biased. On the other hand, there is growing evidence that a longer duration of illness may have a “pernicious effect on physical health, brain development and self-efficacy” (for a review see [41]). Therefore, all approaches, including hospital admission, have to be considered if a patient fails to improve with an outpatient approach.

**Table 1:** Criteria for inpatient care in adolescent AN

1. Insufficient response to outpatient treatment
2. Risk of suicide or severe self-harm
3. Acute medical stabilization necessary
4. Severe social problems or psychiatric comorbidity

In sum, although adolescents seem to benefit from IP more than adults do, the high relapse and readmission rates as well as the high financial costs have challenged the view that IP is the treatment of choice in moderately to severely ill adolescents. In addition, adolescents with AN often do not want to be hospitalized and tend to experience hospitalization as more coercive than adult patients [42]. Consequently, rates of dropout from inpatient treatment programs are up to 25 % [43, 44]. In the French study by Godart [43], dropout was significantly higher when the patient was living with only one parent, had been hospitalized previously, had a lower BMI at admission and was over 18 years [44].

### Day patient treatment (DP)

In the four European guidelines mentioned above, DP is listed as one possible treatment setting “according to appropriate care level” [10]. DP for AN may be an alternative to IP or may be useful as an approach following IP [30]. In addition to potential cost savings associated with DP, the skills that patients obtain while in DP treatment may be more easily transferred to the home setting and thus more generalizable to everyday life. Moreover, compared with IP, DP may be more likely to increase patients’ autonomy and self-confidence. In DP, the family is more involved in managing refeeding, which is a strategy that has been shown to be effective in the treatment of adolescent AN [45, 46]. In addition, during DP, adolescents are able to maintain contact with their peers and to participate in school activities and other social networks, thus supporting social competence. This feature of DP is of considerable importance, as patients with AN are known to have a range of social difficulties [47]. All in all, by keeping patients in touch with the challenges of everyday life, DP may ease the transition from hospital to the outside world and decrease the subsequent risk of rapid relapse.

However, until recently, there were only two small uncontrolled studies from Australia and the US demonstrating preliminary efficacy of a day patient program in younger patients [48]. The authors reported that the 12–18-year-old Australian female patients enrolled in the study benefitted from DP, as evidenced by their significant weight gain and reduction in eating disorder behavior [49]. In a US family-centered day patient program for children with a mean age of 13, participants showed significant improvement in weight and psychological outcomes at discharge. Unfortunately, there was no follow-up assessment in either study [48].

Our own multicenter open-label trial included 172 participants from six treatment centers in Germany with initial admission to hospital for AN. After 3 weeks of inpatient care, the patients were randomly assigned to either IP or DP with an identical treatment program in both settings [35].

At the 12-month follow-up point, DP was not found to be inferior to IP with respect to BMI. In a scale evaluating specific eating disorder and global psychological outcomes (Morgan & Russell Average Outcome score, [50]), the patients in the DP group had better scores in the domains of mental well-being and psychosexual adjustment than the IP group. In addition, health care costs were significantly lower for DP than for IP. The number of serious related adverse events (such as suicidal ideation) was low and similar in both study groups. Thus, we concluded that the use of DP after brief inpatient care was a safe and less costly alternative to IP for adolescent patients with non-chronic AN and that DP should thus be broadly used in the treatment of AN [51]. A follow-up study that shows even more promising results is being prepared.

### Outpatient treatment

If receiving outpatient care, patients should obtain psychological therapy in addition to physical monitoring (NICE guidelines). A description of the most frequently applied psychological treatments in children and adolescents is given below. Most guidelines emphasize that the health care professional should be experienced in the treatment of eating disorders (NICE and German guidelines). The Dutch guidelines primarily recommend a mental health care center with expertise in eating disorders because of the multidisciplinary treatment capabilities.

### Multimodal treatment approach

There is some evidence that a multimodal treatment approach in all types of settings (inpatient, day care, and outpatient settings) is effective in restoring a healthy body weight and better psychological and social functioning in patients with AN [52]. In adolescents, this approach is mainly based on three components (Table 2).

**Table 2:** Main components of multimodal treatment approach in adolescent AN

1. Nutritional rehabilitation and nutritional counseling as well as the treatment of medical problems
2. Family (or parent) counseling or family therapy
3. Individual therapy (and, whenever possible, additional group sessions) to correct dysfunctional thoughts concerning weight and shape

### Nutritional rehabilitation and principles of weight gain

According to most international guidelines, the restoration of a healthy body weight is one of the central goals in the treatment of AN (APA [30]; German and Spanish guidelines; NICE guidelines). Weight gain during inpatient or outpatient treatment and the maintenance of weight gain after discharge from treatment have been proven to be important prognostic factors for the short- and long-term course of adolescent AN [53–55]. From the patient’s perspective, weight gain is a significant predictor of improved psychological outcomes, including global eating disorder psychopathology [56].

However, there is widespread variation in the practice of defining a “healthy body weight”, i.e., the target weight in the treatment of childhood and adolescent AN. In a study by Gowers and coworkers [57], 18 eating disorder services from the UK, Denmark, Finland, the Netherlands, France, Germany, Sweden, Spain, and Ireland provided recommendations for weight targets for a 14-year-old girl. When BMI was used, specifications of target weight ranged from 17.5 to 21 kg/m^2^ and from the 10th to the 75th age-adjusted percentile.

As a rule, a healthy body weight might be defined as the weight at which menstruation reoccurs [30]. Note, however, that this purpose is not mentioned in the four European guidelines. Many patients do not resume menses despite weight restoration at discharge from treatment [58]. Thus, a target weight supported by scientific evidence should be determined by the patient’s therapist. In our recent study including 172 adolescents, achievement of the 15th to 20th age-adjusted BMI percentile at discharge was a significant positive predictor of the resumption of menses at 12-month follow-up [58]. Patients who menstruated had a mean age-adapted BMI percentile of 24 at follow-up, which is very similar to the body weight at the time point of resumption of menses observed by Golden et al. [59] and roughly corresponds to the 95 % EBW (%Expected Body Weight ≙ observed BMI/50th BMI percentile × 100) reported by the Le Grange group [60]. In short, an increase in BMI to the 25th age-adjusted percentile and weight maintenance thereafter are indispensable preconditions for the resumption of menses [8]. Note, however, that premorbid BMI and the weight at which menstruation ceased are also important in determining target weight because there is some evidence from our and from an Italian study that patients with a higher premorbid BMI will only menstruate at a BMI nearer to their original body weight [58, 61, 62]. Younger patients and patients with premenarchal onset of AN are at a particular risk for protracted amenorrhea [58, 63].

In contrast to a strictly defined target weight, some clinicians prefer a weight range of “around” the 25th age-adapted BMI percentile.

### Expected rate of weight gain

European guidelines differ quite substantially in their recommendations for weekly weight gain (Table 3), and the majority does not distinguish between adolescents and adults. Some international authors report that early weight gain in adolescent girls during the first weeks of (outpatient) treatment is positively related to outcomes at the end of treatment but not to outcomes at 12-month follow-up [64].

**Table 3:** Recommendations for weekly weight gain according to 4 European guidelines

NICE guidelines (no modification for adolescents):	0.5–1.0 kg in an inpatient setting, 0.5 kg in an outpatient setting
Dutch guidelines for adolescents:	0.5–1.0 kg in an outpatient setting, between 0.5 and 1.5 kg in a clinical setting and up to 2.0 kg in a somatic clinical setting
Spanish guidelines (no modification for adolescents):	Ponderal weight gain greater than 0.5 kg with up to 1 kg/week
German guidelines:	0.5–1.0 kg (at the most) in an inpatient setting, 0.2–0.5 kg in an outpatient seeing (no strong precept)

As far as we know, clinical guidelines do not define the caloric intake that is necessary to achieve weight gain goals. The NICE guidelines only mention an additional amount of 3500–7000 calories per week without differentiating between in- and outpatient treatment or between adolescents and adults. This range is very large, and it is not specified which patients may benefit from lower or higher caloric intake.

There is a noticeable difference between European and US-American guidelines and their precepts of recommended rates of weight gain. The modest weekly weight gain of 0.5–1.0 kg during AN inpatient treatment practiced in several European countries stands in contrast to the recommendations of the US-American guideline and various American groups [65, 66]. These authors recommend/achieve weight gains of 1–2 kg/week during hospitalization.

### Refeeding practices

Common practice involves beginning the refeeding process cautiously. Inpatient programs often start weight rehabilitation on low-calorie regimens, typically 30–40 kcal/kg body weight/day or even less in severely emaciated patients. Clinicians are afraid of overfeeding their patients with the consequence of cardiac, renal, and neurologic complications (the so-called “refeeding syndrome”). According to a systematic review based on seven observational studies with approximately 400 inpatients, the prescribed energy intake ranges from 1000 to >1900 kcal, with a steady increase during the time of hospitalization [67]. In the department of the first author, we begin refeeding at even lower rates in very emaciated patients, which corresponds to the recommendations by an expert group of the Royal College of Psychiatrists (Junior MARSIPAN http://www.rcpsych.ac.uk/usefulresources/publications/collegereports/cr/cr168.aspx, accessed 25.1.2015), who estimate 20 kcal/kg body/weight/day as safe, but advise less (5–10 kcal/kg/body weight) for individuals with very low initial weight or comorbid somatic disorders.

However, recent studies have not found any difference in the rates of refeeding syndrome between adolescents who were started on a low-calorie diet compared with those on a high-calorie diet [68, 69]. Hypophosphatemia, a major symptom of refeeding syndrome, was instead associated with low BMI at intake but was not related to the amount of calories consumed [68, 70]. O’Connor and Goldin [71] reported the case of a 10-year-old girl with a body weight of 24 kg on a refeeding diet of only 600 kcal/day, who still developed refeeding syndrome. Thus, some authors are concerned that current refeeding practices starting at very low energy levels will postpone weight recovery and worsen outcomes [72] and have pointed out that “a start low, advance slow” regimen could give cause to the “underfeeding syndrome” [73–75].

Healthy girls between 10 and 13 years of age need approximately 50 kcal/kg body weight for their daily energy needs, and adolescent girls should consume 40–45 kcal/kg/body weight [76]. Healthy young women eat approximately 30 kcal/kg/body weight per day (range of 20–40 kcal/kg/day). In contrast, adolescents with AN tend to eat no more than 10–20 kcal/kg/day.

Refeeding syndrome primarily develops during the first fortnight after starting refeeding [72]. Later in the refeeding process, most guidelines recommend a stepwise increase of daily intake such as 200 kcal/day [72]. Our American colleagues suggest a caloric supply up to 60–100 kcal/kg/day (in other words, 2400–4000 kcal for a girl of 40 kg and 3000–6000 kcal for a young woman of 50 kg) [65].

Based on the findings by the Kaye group, patients with the restricting type of AN, which is the most prevalent in children and adolescents, gain weight more slowly than those with the binge/purge type and likely need an even higher amount of calories. There is also an evidence that the excessive exercise often exhibited by patients with AN requires additional calories to gain the same amount of weight [65].

In addition to the rate of weight gain, there is a dearth of knowledge on macro- and micronutrient needs in adolescent patients with AN. Many of them practice vegetarianism with high fiber intake, low fat intake, normal protein levels, and low or normal carbohydrate intake, which often do not meet basal nutritional requirements. Moreover, recommendations for vitamin or mineral requirements despite imminent risk of osteoporosis remain vague (NICE and Spanish guidelines).

In sum, there is no empirical evidence for an optimal weight gain regimen in adolescent AN. Most of the recommendations are solely based on mainstream clinical or expert opinion. Thus, there is an urgent need to gain further knowledge—both quantitatively and qualitatively—of the nutritional demands of AN patients and to find a common approach for optimizing weight restoration practices.

### Family-oriented therapy

All four guidelines emphasize that family members including siblings should be involved in the treatment of adolescent and childhood AN. The Maudsley FBT is the most established treatment model for adolescents with AN, and several RCTs have demonstrated evidence for symptom remission and weight rehabilitation. Compared to all other treatment approaches in AN, it was assigned the highest evidence grade by the NICE guidelines [11]. While the first RCTs were conducted in the UK [77–79], the more recent ones were performed in the US by the Chicago and Stanford groups [17, 80, 81]. FBT is conceptualized as an outpatient treatment for medically stable non-chronic adolescents and is built on the assumption that the family is the most important resource for the adolescent for weight restoration and return to healthy eating patterns. Treatment is divided into several overlapping phases. During the first phase parental strengths are primarily focused on their child´s weight rehabilitation and normalization of eating. Parents take care of the adolescent´s food intake, supervise meals and restrict physical activity and purging behavior. In the second treatment phase—after having achieved a more healthy weight—the adolescent is encouraged to regain responsibility over food and eating. In turn, parents withdraw from the control of eating; their attention will be solicited by more general questions about age-appropriate adolescent life and functioning. During the third phase, this aspect will be even more emphasized with a focus on normal adolescent development and increasing autonomy and on how the eating disorder has disturbed this process. Last, but not least during this last phase, the necessity of relapse prevention strategies will be stressed [46, 82].

FBT was compared to individual adolescent-focused therapy (AFT) in a large outpatient RCT including 121 adolescents. At 6- and 12- month follow-up significantly more patients in the FBT group achieved remission defined as achieving ≥95 % EBW (see above) and eating disorder scores within 1 SD of published means [17]. At 12-month follow-up, 50 % in the FBT group had achieved full remission. However, in this trial BMI at admission was higher than in several European treatment studies in adolescent AN [37, 83, 84]. In addition to psychological treatment patients had approximately weekly medical monitoring visits by experienced physicians, and 17 % were taking psychotropic medication at baseline. Moreover, 45 % of the Lock et al. sample had been hospitalized before randomisation, and 26 % were hospitalized during treatment.

At the 4-year follow-up, more probands in the adolescent-focused group had made additional weight gains and caught up with the FBT group. Although the result was biased by retaining only 65 % of the original probands, AFT did not seem to be less effective in the longer run [64, 73].

In a study from France, a more systemic form of family therapy was investigated as an adjunctive intervention to treatment as usual after inpatient treatment. At the 18-month follow-up, patients who received adjunctive family therapy had a better outcome regarding “general outcome”, BMI and menstrual status [85].

In a very recent trial, FBT was compared to systemic family therapy. At the end of treatment and 1-year follow-up, there were no statistically significant differences regarding body weight and remission rates. FBT was associated with a significantly faster weight gain at the beginning of treatment, less days spent in hospital and lower treatment costs [81]. Patients with more severe obsessive–compulsive symptoms had more benefit from systemic family therapy.

Other family-oriented approaches are underway, such as a parent-focused treatment as well as stress management and skills training programs for parents and carers. In the parent-focused program patients are randomly allocated to either parent-focused or conjoint family treatment on FBT-basis. While the adolescent patient sees a clinical nurse consultant, parents are supported by a mental health clinician; in the conjoint therapy group, the whole family is seen by a mental health clinician [86]. Caregiver skills programs were established because of the finding that parents and carers of individuals with AN suffer from great objective and subjective burden [87]. They include self-help and web-based interventions [88–90]. Preliminary results demonstrate a reduction of negative experience of caregiving and an improvement of depression.

### Individual therapy

Although FBT is the treatment of choice for adolescent AN, several guidelines also recommend individual meetings with the therapist, e.g., the NICE guidelines (“Children and adolescents with AN should be offered individual appointments with a healthcare professional separate from those with their family members or carers”). In our opinion, this statement is important, as many young patients with AN try to protect their parents and may thus be more honest in speaking to a professional therapist.

In many European countries individual psychotherapy plays an important role as treatment modality in adolescent AN, because FBT is under-resourced in many areas, and parents are not always able to accompany their children regularly.

Individual psychotherapy may be provided on an inpatient or outpatient basis. During the early stages of inpatient treatment, a more supportive treatment strategy may be necessary because of the mental consequences of malnutrition, such as extreme rigidity and perseveration appearing as an overarching preoccupation with weight and shape and persistent thinking about calories and food. Somatically (and mentally) sufficiently stabilized patients may start with a more problem-oriented individual psychotherapy. However, there are virtually no controlled studies on specialist treatments in youth. Some studies include mixed samples of adults and adolescents. The psychological interventions investigated have included adolescent-focused therapy (AFT), behavioral therapy, cognitive-behavioral therapy (CBT), cognitive analytic therapy, interpersonal psychotherapy (IPT), body awareness therapy, and specialist supportive clinical management (SSCM), among others. Most data suggest that patients improve with specialist treatment, but none of these individual approaches has been demonstrated as most effective.

AFT was developed at Stanford University and the University of Chicago and is mainly practiced in the US [91, 92]. It is based on a self-psychology model and consists of three treatment phases with an emphasis on adolescent developmental tasks. After establishing a reliable therapeutic relationship, the goal of the first phase is to identify the patient´s problems and psychological deficits and the way how AN serves to protect the patient against developmental challenges. In the second phase, the aim of the therapy is to help the patient to improve self-efficacy and to try to explore school and vocational aims as well as those of social identity and processes [92]. In addition, the patient is encouraged to gain more independence from the parents. In the third phase, typical developmental problems which have to be faced in adolescence are addressed. The therapist supports the patient to cope with them in an age-appropriate manner, thereby improving self-esteem and independence.

AFT was compared to FBT in an outpatient RCT including 121 adolescents. Although more patients achieved full remission in the FBT group at the 12-month follow-up, AFT did not seem to be less effective at the four-year follow-up (for more details see above) [64, 73].

Because of the considerable acceptance of CBT, which has demonstrated notable improvement in patients with BN and other non-restrictive eating disorders, it has been enhanced to become a transdiagnostic treatment (CBT enhanced, CBT-E). There are two recent European studies of CBT-E in adolescents with AN, one with outpatients [93] and one with inpatients [94]. Detailed descriptions of the implementation of CBT-E in adults [95] and adaptation for adolescents [96] have been published. Similar to adults, CBT-E for adolescents consists of three phases: (1) thinking afresh on the current state and maintaining processes of the eating disorder, including analyses of pros and cons; (2) modification of concerns surrounding weight and shape; and (3) maintaining changes and developing strategies to handle setbacks. The only difference from CBT-E in adults is the routine involvement of parents or caregivers [93]. In the first study, outpatients with AN (*n* = 46) were offered 40 sessions of CBT-E treatment over 40 weeks followed by a 60-week post-treatment phase with only minimal therapeutic support. At 60-week follow-up, 28 % of the patients had achieved 95 % EBW and a substantial amelioration of the eating disorder psychopathology (EDE scores <1 SD above the community mean in 76 % of all patients). Two-thirds of the original sample completed the treatment.

In a second study, CBT-E was adapted for inpatients [94]. A total of 27 adolescents participated in the study, and 26 completed the treatment. There was a significant increase in BMI percentile from the 3rd to the 30th percentile and a considerable decrease in specific and global eating disorder psychopathology. However, in both studies, there was no control group and no comparison with any other specific treatment for adolescents, including family-based treatment.

SSCM combines the principles of supportive psychotherapy and clinical management delivered by a clinician experienced in the treatment of AN, as it is often practiced in general practice or general psychiatric or pediatric hospitals. In addition to the regular monitoring of weight gain, the patient receives nutritional education and advice, and a target weight is established during treatment. Moreover, clinical management includes several components of psychoeducation, such as explanations about the symptoms and course of the eating disorder. These interventions are embedded in a reliable and supportive relationship between the clinician and patient to foster an environment of empathy and acceptance [52, 97].

Older adolescents were included in the first SSCM study, and the outcomes were compared to those for IPT and CBT. While SSCM was superior at the end of treatment, the groups no longer differed at 7-year follow-up with respect to physical and cognitive measures [97, 98].

### Cognitive remediation therapy

Studies have shown that individuals with eating disorders have inefficiencies in neuropsychological functioning, [99–101]. It has been suggested that neuropsychological deficits preexist and underlie the etiology of eating disorders development and relapse. Neuropsychological deficits in eating disorders are set-shifting and weak central coherence. Set-shifting concerns the ability to move back and forth between multiple tasks, operations or mental sets, and represents cognitive flexibility. Weak central coherence refers to superior detail processing and weak global integration. Although both deficits have been demonstrated in adults with AN and BN, the picture in adolescents is not so clear. Studies range from firm neuropsychological deficits [102] to subtle deficits [103] or a mixed picture [104] with a subgroup of adolescent patients clearly showing an impaired cognitive profile [105], that may improve after renutrition [106]. A recent systematic review, specifically on set-shifting abilities in children and adolescents, found no overall differences between adolescents with AN and healthy controls in relation to commonly used set-shifting tasks [107], suggesting that these may be indicative of more prolongued illness duration or starvation.

Cognitive remediation therapy (CRT) was developed with the aim of improving cognitive flexibility and thereby improving functioning. The focus of CRT is on *how* patients think, rather than on *what* patients think. It is hypothesized that CRT training works by proliferating and refining neural connections and by teaching new, adaptive strategies, thus making individuals more flexible in the way they think and behave [108]. Preliminary results show small to medium effects of CRT on various measures of cognitive flexibility in adolescents, either presented in groups or individual settings, as an adjunct to other treatments [102, 109].

### Medication

Pharmacotherapy has a very limited evidence base and should not be used as the primary or sole treatment strategy. This view is held by the NICE and the Spanish and German guidelines. In AN, atypical antipsychotics and selective serotonin inhibitors (SSRI) are the most extensively evaluated medications. In recent RCTs and a systematic meta-analysis from the US in adults and adolescents, adjunctive treatment with second-generation antipsychotics did not yield important effects on weight-related outcomes or on eating disorder-specific psychopathology.

In a double-blind placebo-controlled study on risperidone, 40 adolescent patients were randomized to receive either risperidone or placebo in addition to the normal eating disorder program. After 9 weeks, there were no significant differences between the treatment groups in weight gain or (with the exception of “interpersonal distrust”) in eating disorder psychopathology. There were no adverse effects, with the exception of a prolactin increase in the risperidone group [110, 111]. In another placebo-controlled study of 20 adolescent females (only 15 of whom completed the study), the percentage change in median body weight did not differ between the two treatment groups. A trend of increasing fasting glucose levels and insulin levels was found in those individuals medicated with olanzapine [112]. Although both studies lack power because of the small sample size, there was no major effect on weight development and general or specific psychopathology. In a recent systematic review and meta-analysis of the effects of olanzapine, risperidone, and amisulpride in both adolescent and adult samples, no significant increase in BMI or decrease in the drive for thinness and body dissatisfaction was found ([113]; for more details, see Herpertz-Dahlmann [114]). In sum, given the uncertainty of effectiveness and several adverse effects, the recently observed increase in the use of second-generation antipsychotics in AN treatment in the US is difficult to understand [115].

We are well aware that use of medication, particularly of olanzapine, might be necessary intermittently in highly anxious and agitated patients, who otherwise would resist to refeeding [114, 72]. According to the Dutch guidelines, if medication is initiated, “olanzapine is the drug of first choice in treating AN in children and adolescents.” Usually, side effects are mild. Note, however, that all guidelines point to possible adverse effects of atypical antipsychotics, such as prolonged or abnormal QTc interval, and metabolic changes, such as hyperglycemia and hyperlipidemia. It is unclear whether very underweight patients with AN are at a particular risk for developing extrapyramidal symptoms (EPS).

Furthermore, there is no clear evidence of whether and under what conditions SSRI may be beneficial in the treatment of AN. Primarily fluoxetine has been examined during the weight restoration process and the weight maintenance phase of therapy. In two early studies, no effect on weight gain was found [116, 117]. In a study of relapse prevention, 93 weight-restored patients including females 16 years or older were enrolled in a controlled multicenter study combining CBT and pharmacotherapy with fluoxetine. As an adjunctive medication, fluoxetine was not more effective than the placebo, even for depressive and anxiety symptoms [118]. Dutch and German guidelines indicate that the treatment of depression, obsessive–compulsive symptoms, or anxiety with SSRI may be helpful after weight restoration but not during the starvation stage. Weight rehabilitation itself has substantial “antidepressive” and “antiobsessive” effects that should be observed before initiating pharmacotherapy.

Future studies will hopefully illuminate predictors of treatment response or identify different phenotypes of AN who might benefit from psychopharmacological treatment. Novel pharmaceutical substances targeting hormonal circuits of appetite control and food intake may also be important agents in the future treatment of AN [119]. At present, medication should not be the first line treatment, should never be used as the only treatment and always has to consider the patient´s nutritional status.

### Estrogen supplementation

Our guidelines referring to estrogen administration are no longer up to date. The NICE and German guidelines advise not to treat children and adolescents with estrogens because of premature fusion of epiphyses. However, there is no empirical evidence that very low doses of ethinylestradiol used in contraceptives lead to premature fusion of epiphysis and thus to short stature.

The Spanish guidelines suggest that “the need to prescribe estrogens to prevent osteoporosis should be carefully assessed, given that this medication can hide the presence of amenorrhea”. However, estrogen administration alone would not be followed by regular menstruation.

Recently, it has become clear that sex hormone replacement therapy, most commonly administered as oral contraceptives containing estrogen/progesterone, does not prevent or improve the loss of bone mineral density in girls or young women with AN. On the contrary, the regular bleeding that occurs with oral contraceptives is misunderstood as the normalization of the menstrual cycle, although it only indicates endometrial hormone withdrawal bleeding. Moreover, the etiology of osteoporosis/osteopenia in AN bears no physiologic resemblance to that of menopausal women. In a systematic review of the literature, the majority of studies did not find any benefit of oral contraceptives for bone mineral density in AN [120]. Moreover, there is some evidence that the intake of oral contraceptives might even worsen bone mineral density. Among others, low levels of insulin-like growth hormone factor (IGF-1), which mediates many of the growth hormone effects on bones, is a basic cause of reduced bone mass density in AN. There is some evidence that oral estrogen administration is associated with the suppression of IGF-1 production by the liver and of androgen levels, which are also essential for bone development [121, 122]. In a recent randomized trial by Misra et al. [123], transdermal physiologic estrogen replacement was investigated to avoid the use of oral intake. The adolescent sample was divided into mature girls with a bone age ≥15 years and immature girls with a bone age <15 years, who received developmentally adapted doses of estrogen. In comparison to the placebo group, spine and hip bone density increased in the group treated with transdermally administered estrogen [123]. Although this study holds some promise in improving bone mineral density in adolescent AN, much more research is needed. However, all researchers agree that the greatest increase in BMD occurs with the normalization of weight and the resumption of menses.

### Compulsory treatment

In life-threatening AN, involuntary hospitalization and compulsory refeeding may sometimes be necessary to save the person’s life. In the UK, NICE [11] guidance recommends that ‘feeding against the will of the patient should be an intervention of last resort in the care and management of AN’. Further, it is recommended that this ‘is a highly specialized procedure requiring expertise in the care and management of those with severe eating disorders and the physical complications associated with it’ and lastly, that there needs to be a clear legal basis for this action and that the relevant mental health legislation and associated procedures are followed. A recent EU report discusses criteria, standards, and differences in legal frameworks for involuntary placement and treatment in different member states (http://fra.europa.eu/sites/default/files/fra_uploads/2130-FRA-2012-involuntary-placement-treatment_EN.pdf).

Two recent systematic reviews [124, 125] have summarized the limited available research evidence on compulsory admissions and treatment in AN. It is clear from this that these cases tend to be complex with severe ED symptoms and high levels of psychiatric comorbidity. While in the short-term compulsory treatment is often life saving, the longer term effects of this are uncertain. Importantly, qualitative studies suggest that many patients are grateful after the event for having been detained and treated against their will.

*In summary*, the standing of a field is considerably based on its evidence for treatment [12]. Although the number of RCTs and well-designed studies have increased in the recent past, the evidence base for treatment of adolescent AN is still limited. Although most clinicians believe that a multidisciplinary and multimodal treatment approach is the most effective strategy for restoration of healthy body weight and a fundamental change in overvaluation of weight and shape, these recommendations are primarily based on mainstream expert opinion with little empirical standing. Because of the relative rarity of the disorder research should be performed at multiple treatment sites and might connect different European countries. When we look for advances in treatment, a more “brain-directed therapy” [126] emerging from new insights into genetics, neurobiology, and cognitive functioning of eating disordered patients might help us to develop more effective and individualized strategies to overcome this often serious and disabling disease.

### Key points

1. Recent changes of classification criteria in DSM-5 facilitate a diagnosis of AN in younger subjects, but weight criteria and starvation-induced physical signs still remain undefined.
2. Practice guidelines from the four European countries Germany, Spain, The Netherlands, and the UK correspond in major recommendations for the treatment of AN, but there is no convention, which intensity of treatment (inpatient, day patient, outpatient) is necessary at different stages of the illness.
3. There is no consensus and no evidence—based recommendation about the most effective way to achieve nutritional rehabilitation and whether and how a target weight should be set.
4. Although FBT is recommended as the treatment of choice in adolescent AN, there is a dearth of European studies on availability and effectiveness.
5. There is an urgent need for further common European research efforts to establish more evidence-based clinical guidelines and recommendations for practice areas in the treatment of adolescent AN.
